# Supplementary material for: Transcriptome Analysis Revealed the Potential Molecular Mechanism of Anthocyanidins’ Improved Salt Tolerance in Maize Seedlings
Source: Plants (Basel). 2023 Jul 27;12(15):2793. doi: 10.3390/plants12152793 (PMC10421157; doi:10.3390/plants12152793)
Supplement: Supplementary file 1 [file plants-12-02793-s001.zip › Supplementary figures-1-5 .pdf]

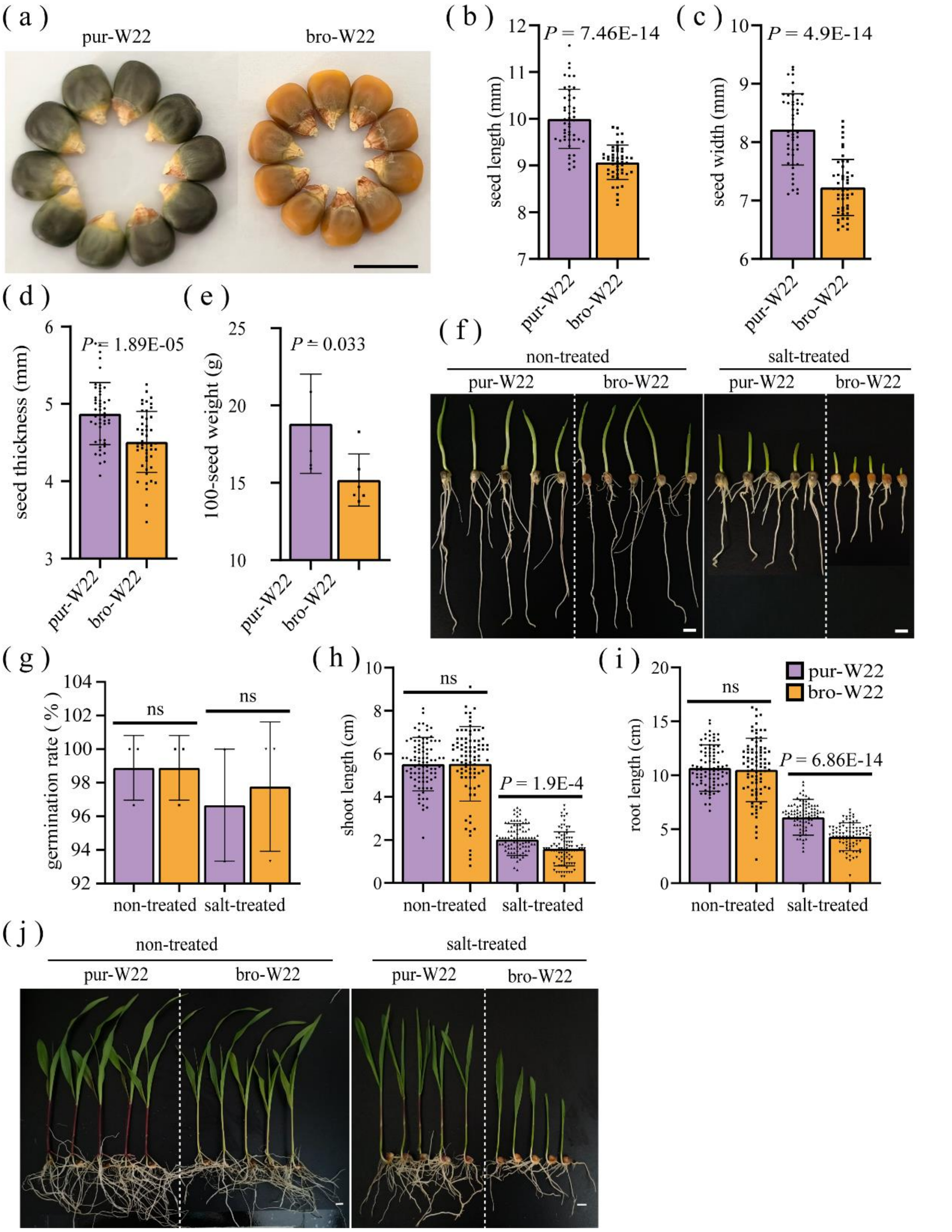

Figure S1. Characteristic analysis of seeds and seedlings for pur-W22 and bro-W22. (a-e) Seeds phenotypes of pur-W22 and bro-W22 (a), seed length (b), seed width (c), seed thickness (d) and 100-seed weight (e). (f-i) seedling phenotypes (f), seed germination rate (g), shoot length (h) and root length (i) of pur-W22 and bro-W22 grown for 7 days under non-treated and salt-treated (100 mM NaCl solution). Bar = 1 cm, black dots represent individual values, *P* values calculated by one-way ANOVA, $P < 0.05$ represents a significant difference, $P < 0.01$ represents the difference is extremely significant, ns represents no difference. (j) seedling phenotypes of pur-W22 and bro-W22 grown for 14 days under non-treated and salt-treated.

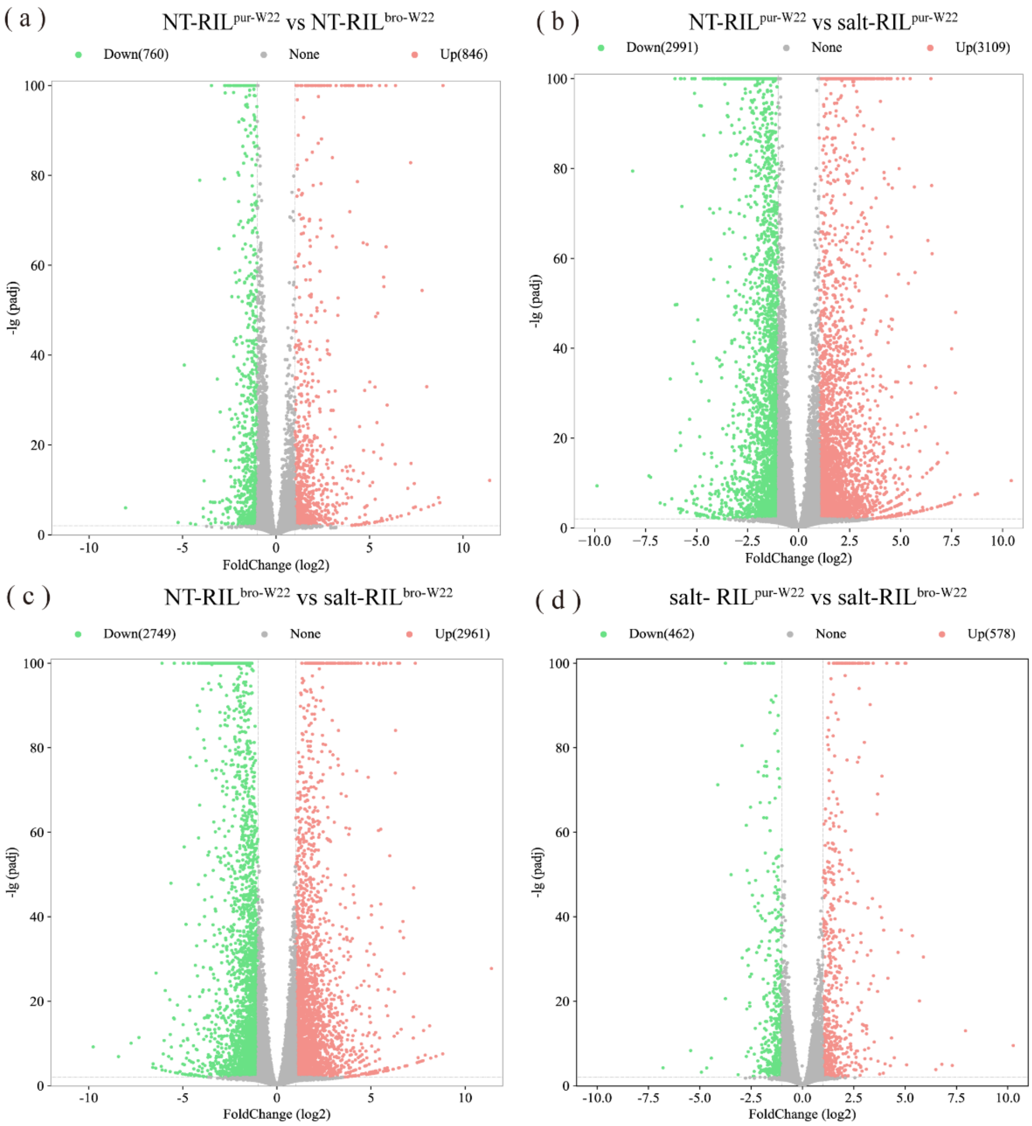

Figure S2. The volcano plots of differentially expressed genes (DEGs) in NT-RIL$^{\text{pur-W22}}$ vs NT-RIL$^{\text{bro-W22}}$ (a), NT-RIL$^{\text{pur-W22}}$ vs salt-RIL$^{\text{pur-W22}}$ (b), NT-RIL$^{\text{bro-W22}}$ vs salt-RIL$^{\text{bro-W22}}$ (c) and salt-RIL$^{\text{pur-W22}}$ vs salt-RIL$^{\text{bro-W22}}$ (d) groups.

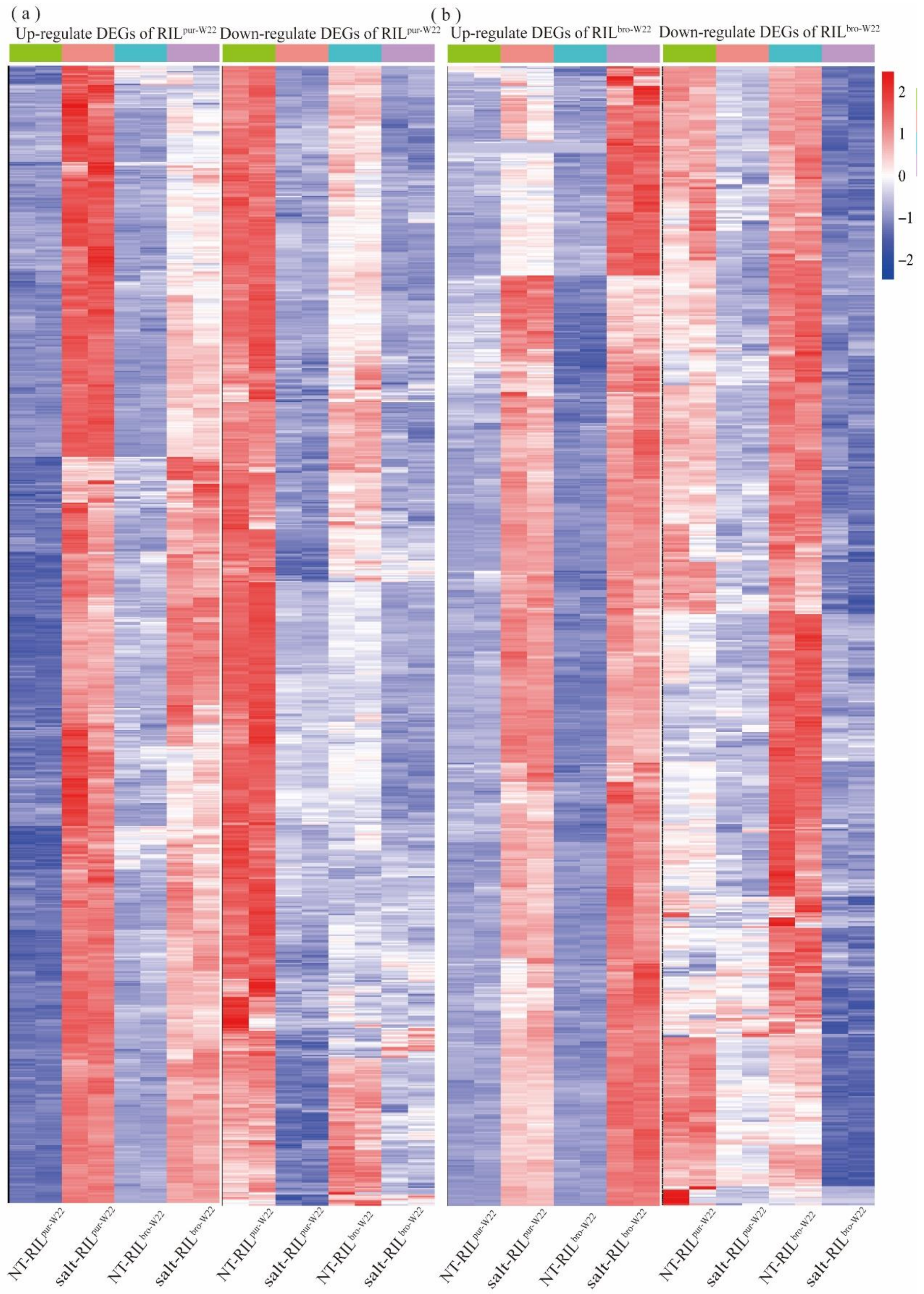

Figure S3. Heatmap cluster of DEGs which specifically expressed in RIL$^{pur\text{-}W22}$ (a) and RIL$^{bro\text{-}W22}$ (b) seedlings to response the salinity stress. The left side shows up regulated DEGs, and the right side shows down regulated DEGs. Each sample has two compartments, which are two biological replicates. According to the standardized FPKM, red and blue indicate high and low abundance, respectively.

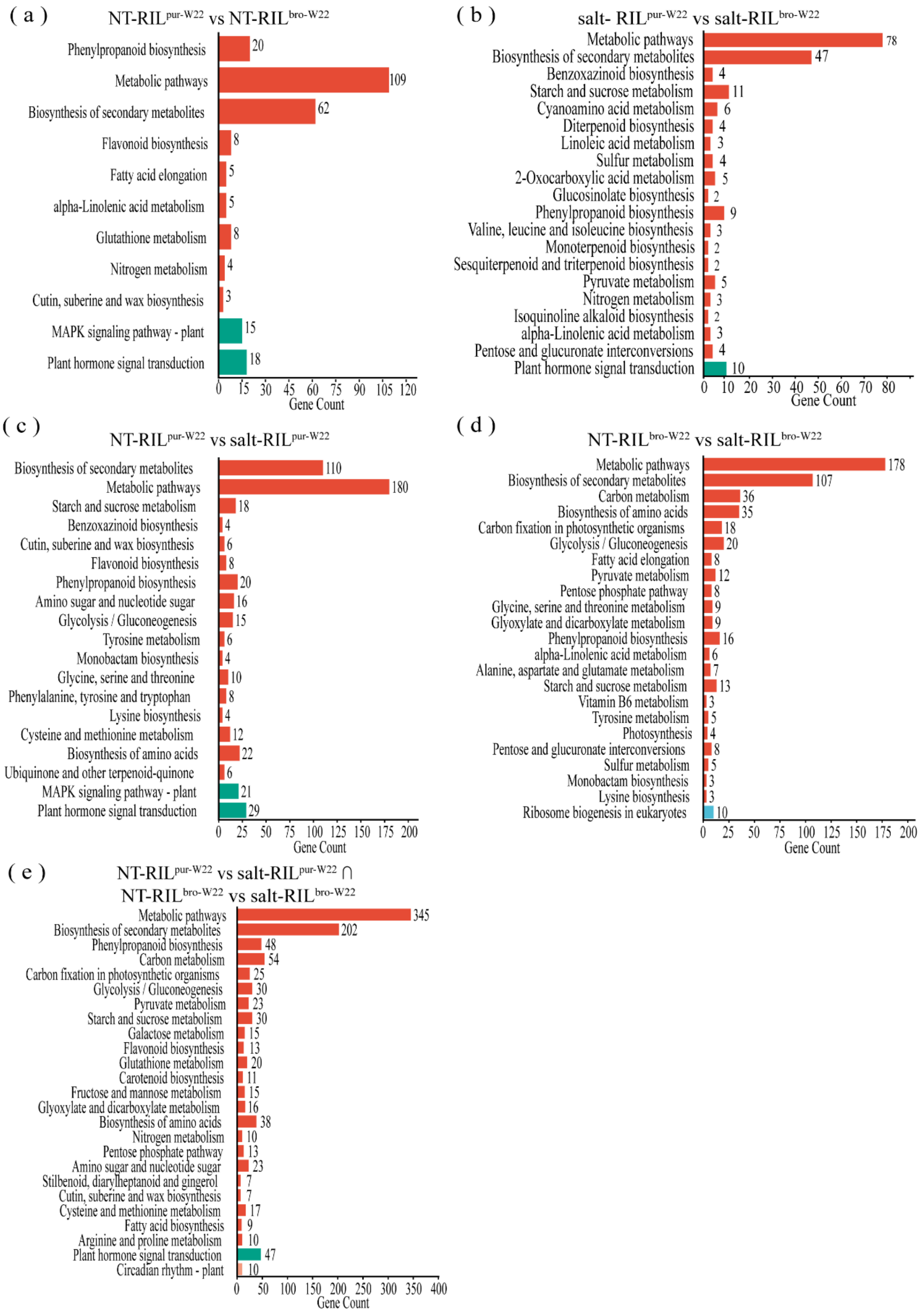

Figure S4. KEGG analysis of DEGs in NT-RIL$^{pur\text{-}W22}$ vs NT-RIL$^{bro\text{-}W22}$ (a), salt-RIL$^{pur\text{-}W22}$ vs salt-RIL$^{bro\text{-}W22}$ (b), NT-RIL$^{pur\text{-}W22}$ vs salt-RIL$^{pur\text{-}W22}$ (c), NT-RIL$^{bro\text{-}W22}$ vs salt-RIL$^{bro\text{-}W22}$ (d) and NT-RIL$^{pur\text{-}W22}$ vs salt-RIL$^{pur\text{-}W22}$ ∩ NT-RIL$^{bro\text{-}W22}$ vs salt-RIL$^{bro\text{-}W22}$ (e) groups.

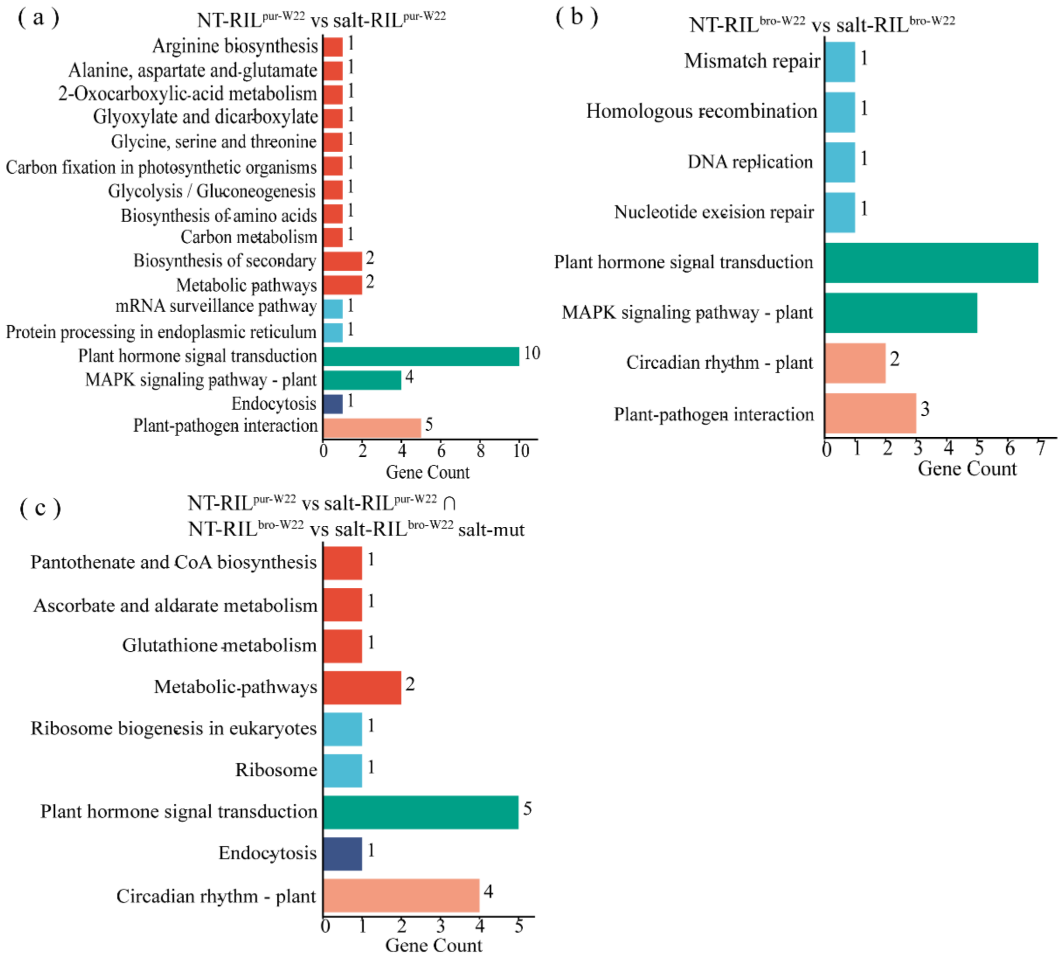

Figure S5. KEGG analysis the DEGs encoding transcription factors in NT-RIL$^{pur\text{-}W22}$ vs salt-RIL$^{pur\text{-}W22}$ (a), NT-RIL$^{bro\text{-}W22}$ vs salt-RIL$^{bro\text{-}W22}$ (b) and NT-RIL$^{pur\text{-}W22}$ vs salt-RIL$^{pur\text{-}W22}$ ∩ NT-RIL$^{bro\text{-}W22}$ vs salt-RIL$^{bro\text{-}W22}$ (c) groups.
